# Sunitinib but not VEGF blockade inhibits cancer stem cell endothelial differentiation

**DOI:** 10.18632/oncotarget.3123

**Published:** 2015-02-28

**Authors:** Alessia Brossa, Cristina Grange, Letizia Mancuso, Laura Annaratone, Maria Antonietta Satolli, Massimiliano Mazzone, Giovanni Camussi, Benedetta Bussolati

**Affiliations:** ^1^ Department of Molecular Biotechnology and Health Sciences, University of Torino, Torino, Italy; ^2^ Department of Medical Sciences, University of Torino, Torino, Italy; ^3^ Department of Oncology, University of Torino, Torino, Italy; ^4^ Laboratory of Molecular Oncology and Angiogenesis, Vesalius Research Center, KU Leuven, 3000 Leuven, Belgium; ^5^ Laboratory of Molecular Oncology and Angiogenesis, Vesalius Research Center, VIB, 3000 Leuven, Belgium

**Keywords:** tumor stem cells, VEGF, HIF, angiogenic therapy, angiogenesis

## Abstract

Different mechanisms of angiogenesis and vasculogenesis are involved in the development of the tumor vasculature. Among them, cancer stem cells are known to contribute to tumor vasculogenesis through their direct endothelial differentiation. Here, we investigated the effect of anti-angiogenic therapy on vasculogenesis of cancer stem cells derived from breast and renal carcinomas. We found that all the anti-angiogenic approaches impaired proliferation and survival of cancer stem cells once differentiated into endothelial cells *in vitro* and reduced murine angiogenesis *in vivo*. At variance, only VEGF-receptor inhibition using the non-specific tyrosine kinase inhibitor Sunitinib or the anti-VEGF-receptor 2 neutralizing antibody, but not VEGF blockade using Bevacizumab, impaired the process of endothelial differentiation *in vitro*, suggesting a VEGF-independent mechanism. In addition, tyrosine kinase inhibition by Sunitinib but not VEGF blockade using the soluble VEGF trap sFlk1 inhibited the cancer stem cell-induced vasculogenesis *in vivo*. Accordingly, Sunitinib but not Bevacizumab inhibited the induction of hypoxia-inducible factor pathway occurring during endothelial differentiation under hypoxia. The present results highlight a differential effect of VEGF-receptor blockade versus VEGF inhibition in tumor vascularization. VEGFR blockade inhibits the process of tumor vasculogenesis occurring during tumor hypoxia whereas the effect of VEGF inhibition appears restricted to differentiated endothelial cells.

## INTRODUCTION

Tumor vascularization is a fundamental step for tumor growth, expansion and progression [1]. Recent data indicate that the tumor vasculature is quite heterogeneous possibly due to different mechanisms of angiogenesis and vasculogenesis [2]. Tumor cells can secrete growth factors and cytokines in order to recruit normal endothelial cells from adjacent vessels [3, 4]. Moreover, tumor endothelial cells may derive from an intra-tumor embryonic-like vasculogenesis due to differentiation of normal stem cells of hematopoietic origin [5]. Finally, tumor cells with stem cell properties, so called cancer stem cells (CSC), of different solid tumors may participate to tumor vasculogenesis by a direct endothelial differentiation [6–16]. The role of CSC vasculogenesis is emerging as an important mechanism for tumor progression, and selective targeting of endothelial cells generated by CSC in xenografted tumors was recently showed to induce tumor reduction and degeneration [14]. We previously isolated CSC and deriving clones from breast and renal carcinomas that were able to differentiate into both epithelial and endothelial cells *in vitro* [15, 16]. I*n vivo*, breast and renal CSC and deriving clones generated epithelial tumors as well as tumor vessels, indicating that at least a fraction of tumor vessels derived from the endothelial differentiation of CSC [15, 16].

Drugs that target tumor vascularization have been recently introduced in the clinical practice for different solid tumors. Anti-angiogenic therapies may directly target endothelial cells in the growing vasculature, as tyrosine kinase inhibitors do, or indirectly block the activity of angiogenesis inducers such as VEGF. In particular, the multi-tyrosine kinase inhibitors Sunitinib and Sorafenib directly target VEGF receptors (VEGFRs) and other non-endothelial receptors such as CD117 and the receptors for Platelet Derived Growth Factor (CD140), for Colony Stimulating Factor 1 and for Glial cell line-Derived Neurotrophic Factor (RET) [17]. In addition, the anti-VEGF mAb (Bevacizumab) potently binds to VEGF preventing its docking with VEGFRs [18].

These anti-angiogenic drugs are known to affect all the different VEGF-dependent mechanisms involved in the angiogenic process, such as endothelial cell proliferation, survival and vessel stabilization [4]. However, the effect of these drugs on alternative strategies of tumor vascularization, and in particular on the CSC-derived endothelial cells and on the process of their differentiation, is at present unknown.

In the present study, we set up a model of hypoxia-induced endothelial differentiation of CSC from breast and renal carcinomas and we aimed to investigate the role of the anti-angiogenetic drugs Sunitinib and Bevacizumab both on CSC-derived differentiated endothelial cells and on the hypoxia-induced process of CSC differentiation into endothelial cells. Moreover, we investigated the effect of Sunitinib and of the soluble VEGF trap sFlk1 on CSC-induced vasculogenesis *in vivo*.

## RESULTS

### Endothelial differentiation of breast and renal CSC under hypoxia

**Figure 1:**
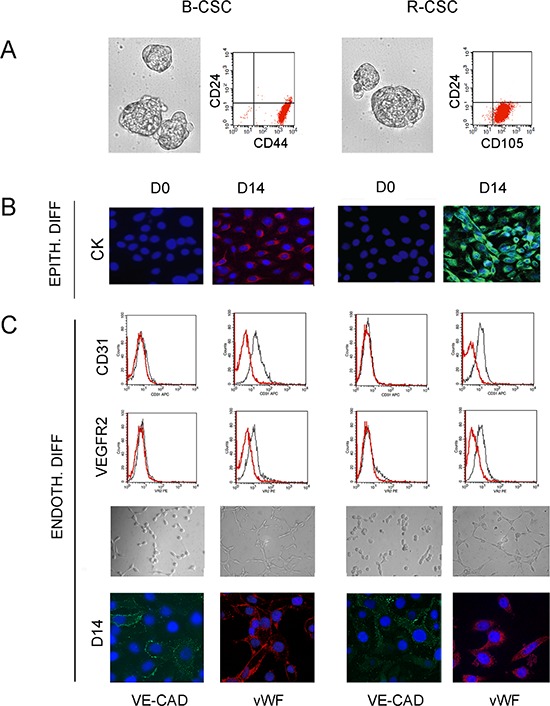
Characterization and differentiative properties of CSC from breast and renal carcinomas **Panel A** and **B**. B-CSC and R-CSC grew in spheres and were characterized as CD24^−^/CD44^+^ or CD24^−^/CD105^+^ cells, respectively **(A)**. B-CSC and R-CSC lacked cytokeratin (CK) that was acquired when cultured in epithelial differentiating conditions (EPITH. DIFF.) for 14 days (D14), as compared with basal condition (D0) **(B)**. **Panel C**. B-CSC and R-CSC cultured for 14 days (D14) in endothelial differentiating conditions under hypoxia (ENDOTH. DIFF.) acquired the endothelial-specific markers CD31, VEGFR2, VE-cadherin (VE-CAD) and vWF and the ability to organize into capillary-like structures. Original magnification: immunofluorescence staining: x400; tubulogenesis: x200. Nuclei were counterstained with Hoechst dye.

We previously isolated and characterized CSC from renal and breast carcinomas showing tumor-initiating and differentiative ability *in vitro* and *in vivo* [15, 16 and Supplementary Table 1]. As reported, B-CSC were able to grow in mammospheres, were CD44^+^/CD24^−^ and showed absence of differentiation markers of the cell types of the glandular epithelium as they did not express cytokeratin-14 and -18 [16] (Figure 1A and 1B). In analogy, CSC from renal carcinomas were identified as CD105^+^ CSC clones, grew in spheres and lacked expression of epithelial differentiative markers such as cytokeratin [15] (Figure 1A and 1B). Both B-CSC and R-CSC were able to differentiate into epithelial cells, as shown by the acquisition of cytokeratin *in vitro*, when cultured in RPMI plus 10% FCS (Figure 1B). In addition, they acquired endothelial cell markers when cultured for 14 days in the presence of a complete medium containing VEGF and 10% FCS [15, 16]. The absence of contaminating cells is supported by the clonal origin of the CSC lines. In the present study, to mimic the tumor microenvironment, we set up a protocol of endothelial differentiation under hypoxia (1% O_2_) in the absence of growth factors or serum. CSC cultured under this condition showed the ability to differentiate *in vitro* into endothelial cells. CSC acquired, after 14 days of endothelial differentiation, full expression of endothelial markers such as CD31, VEGFR2, VE-cadherin, vWF (Figure 1C) and the ability to organize into capillary-like structures (Figure 1C).

### Anti-proliferative and cytotoxic effect of Sunitinib and Bevacizumab on CSC-deriving endothelial cells

**Figure 2:**
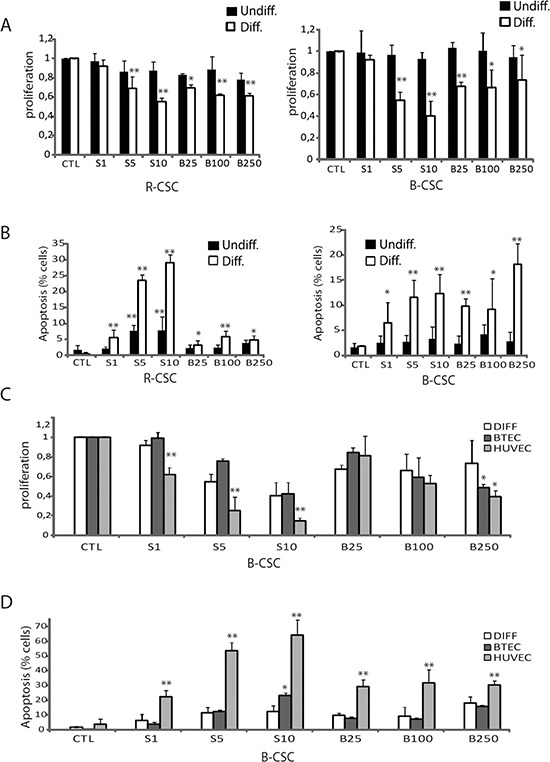
Cytotoxic effect of Bevacizumab and Sunitinib on CSC-derived endothelial cells **Panel A** and **B**. Effect of 1–10 μM Sunitinib (S1-S10) and of 25–250 μg/ml Bevacizumab (B25-B250) on proliferation **(A)** and apoptosis **(B)** of B-CSC and R-CSC before (Undiff, black columns) and after the endothelial differentiation (Diff., white columns). **Panel C** and **D**. The effect of Bevacizumab and Sunitinib on endothelial differentiated CSC was compared to that on total breast tumor-derived endothelial cells (BTEC) or on normal endothelial cells (HUVEC). Data are mean ± SD of five different experiments (**A** and **B**) or three different experiments **(C** and **D)**. Student's *t* test was performed: **= *p* < 0.001, *= *p* < 0.05 drug treated vs CTL cells.

We evaluated the effect of the anti-angiogenic drugs Sunitinib and Bevacizumab on CSC and CSC-derived endothelial cells. No effect of Sunitinib and Bevacizumab was observed on the proliferation of undifferentiated B-CSC and R-CSC (Figure 2A). Indeed, these cells did not express the growth factor receptors known to be target of Sunitinib (VEGFR1, 2 and 3, CD117, CD140; not shown). A slight but significant cytotoxic effect was observed on R-CSC at 5–10 μM Sunitinib, possibly related to a toxic drug effect (Figure 2B), as previously reported on renal cancer cells at doses higher than 5 μM (17). At variance, Sunitinib (5–10 μM) and Bevacizumab (25–250 μg/ml) significantly impaired proliferation of endothelial-differentiated CSC (Figure 2A). In addition, Sunitinib (1–10 μM) and Bevacizumab (25–250 μg/ml) significantly reduced their survival (Figure 2B). This is possibly due to the acquisition by differentiated cells of the expression of VEGFRs (Figure 1C) and not of CD117 or CD140; not shown. We also tested whether the response to these drugs on proliferation and survival was comparable to that of the total endothelial cell population derived from a breast tumor (BTEC) and of normal endothelial cells (HUVEC). The effect observed on endothelial-differentiated B-CSC was comparable to that of BTEC. In contrast, HUVEC showed a higher sensitivity to the anti-proliferative and cytotoxic effects of these drugs (Figure 2C and 2D).

### Effect of sunitinib but not of bevacizumab on endothelial differentiation of CSC *in vitro*

**Figure 3:**
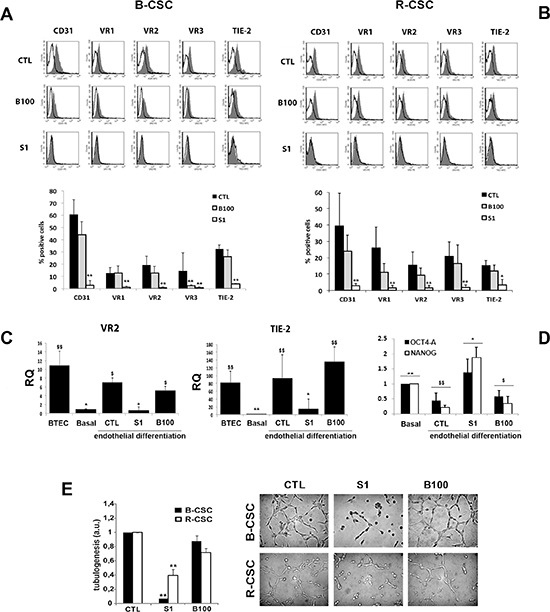
Effect of Bevacizumab and Sunitinib on the endothelial differentiation of CSC **Panel A** and **B**. 1 μM Sunitinib (S1), but not 100 μg/ml Bevacizumab (B100), impaired the hypoxia-mediated endothelial differentiation of B-CSC **(A)** and R-CSC **(B)** as shown by the lack of acquisition of endothelial specific markers. In the representative FACS analyses, the grey area shows binding of the specific antibody and the dark line the isotypic control. In the lower histogram, the percentage of expression is reported. Data are mean ± SD of five different experiments. Student's *t* test was performed: **= *p* < 0.001, *= *p* < 0.05 vs CTL. **Panel C**. Quantitative RT-PCR analysis showing the acquisition of the expression of endothelial markers VEGFR2 (VR2) and TIE-2 by B-CSC after endothelial differentiation (CTL) in respect to undifferentiated B-CSC (Basal). Sunitinib (1 μM, S1) but not Bevacizumab (100 μg/ml, B100) abrogated VEGFR2 and TIE-2 mRNA expression. Total breast tumor-derived endothelial cells (BTEC) were used as positive control of differentiation. Data were normalized to GAPDH mRNA and to 1 for undifferentiated CSC (Basal) and expressed as relative quantification (RQ). Data are mean ± SD of three different experiments. ANOVA with Newmann-Keuls' multicomparison test was performed: *= *p* < 0.05 and **= *p* < 0.001 vs CTL; $ = *p* < 0.05 and $$= *p* < 0.001 vs Basal. **Panel D**. Quantitative RT-PCR analysis showing the reduction of Oct4-A and Nanog by CSC differentiated into endothelial cells (CTL) in respect to undifferentiated CSC (Basal). CSC differentiated in the presence of 1 μM Sunitinib (S1), but not of 100 μg/ml Bevacizumab (B100) maintained these markers. Data were normalized to GAPDH mRNA and to 1 for Basal and expressed as relative quantification (RQ). Data are mean ± SD of three different experiments. ANOVA with Newmann-Keuls' multicomparison test was performed: *= *p* < 0.05 and **= *p* < 0.001 vs CTL; $ = *p* < 0.05 and $$ = *p* < 0.001 vs Basal. **Panel E**. Effect of Bevacizumab and Sunitinib on endothelial-differentiated CSC organization into capillary-like structures. Quantitative evaluation and representative micrographs show the formation of capillary-like structures by control cells (CTL) and by cells treated with 1 μM Sunitinib (S1) or 100 μg/ml Bevacizumab (B100). Data are expressed as the mean ± SD of the length of capillary-like structures, evaluated by the computer analysis system in arbitrary units (a.u.) in at least 10 different fields. Four different experiments per group were carried out in duplicate. Original magnification x200. Student's *t* test was performed: **= *p* < 0.001 vs CTL.

We next investigated whether a chronic treatment with the anti-angiogenic drugs could impair the endothelial differentiative ability of B-CSC and R-CSC in hypoxia. For these experiments, the dose of 1 μM Sunitinib and 100 μg/ml Bevacizumab was selected as non-toxic on CSC. As shown in figure 3A and 3B, Sunitinib almost completely abolished the acquisition of the endothelial markers CD31, VEGFR1, 2 and 3 and Tie-2 by both B-CSC and R-CSC after 14 days in differentiating conditions. In contrast, cells treated with Bevacizumab maintained the endothelial differentiative ability as shown by the acquisition of all endothelial markers (Figure 3A and 3B). This was also confirmed at mRNA level, as VEGFR2 and Tie-2 mRNA expression, evaluated by RT-PCR after endothelial differentiation, was comparable to that in total tumor endothelial cells (BTEC) (Figure 3C). The endothelial markers were lacking in Sunitinib treated CSC, and not in Bevacizumab treated cells (Figure 3C), further supporting the cytofluorimetric data. In parallel, in the presence of Sunitinib, but not of Bevacizumab, CSC maintained the expression of the stem markers Oct4-A and Nanog, downregulated by control cells after endothelial differentiation (Figure 3D). Moreover, CSC cells differentiated in the presence of Sunitinib did not acquire the endothelial ability to organize in tubular-like structures (Figure 3E), confirming that Sunitinib inhibited the endothelial property of CSC. At variance, CSC differentiated in the presence of Bevacizumab acquired the ability to organize in tubular structures, as control CSC (Figure 3E).

### Dispensable role of VEGF in endothelial differentiation of CSC

**Figure 4:**
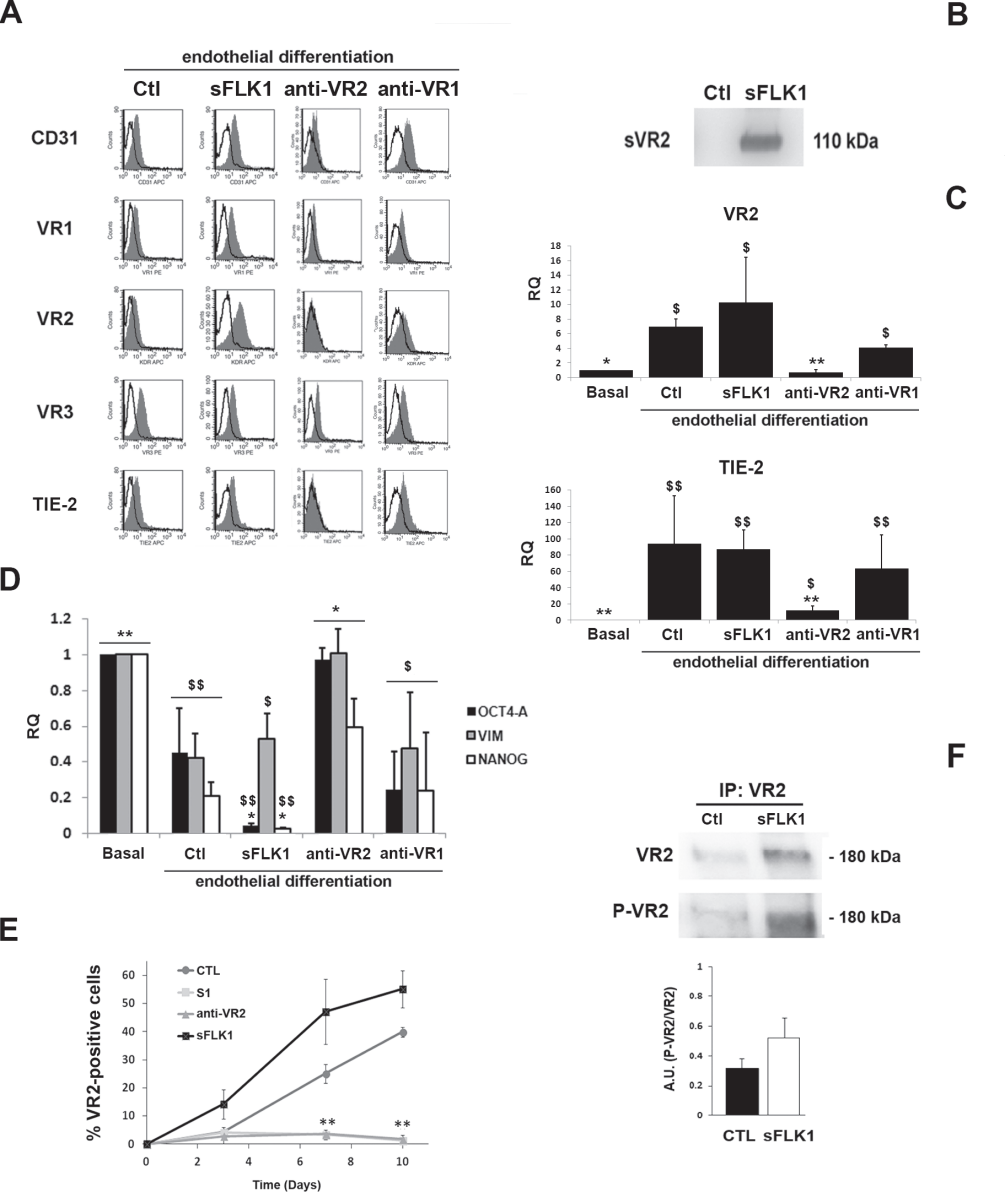
Effect of the VEGF trap sFlk1 and VEGFR blockade on CSC endothelial differentiation **Panel A**. Representative FACS analyses showing the expression of endothelial differentiation markers by B-CSC infected with lentiviruses carrying an empty vector (Ctl) or the soluble VEGF trap sFlk1 (sFLK1 cells) or treated with an anti-VEGFR2 and an anti-VEGFR1 neutralizing Abs (anti-VR2 and anti-VR1). The grey area shows binding of the specific antibody and the dark line the isotypic control. **Panel B**. Western blot micrograph showing the presence of the soluble VEGFR2 (sVR2) in the supernatant of cells expressing the soluble VEGF trap sFlk1 (sFLK1 cells) but not an empty vector (Ctl). **Panel C**. Quantitative RT-PCR analyses showing the acquisition of the expression of VR2 and TIE-2 after 14 days of endothelial differentiation by Ctl, sFLK1 B-CSC and by B-CSC incubated with the anti-VR1, but not by B-CSC incubated with the anti-VR2, during differentiation process. Data were normalized to GAPDH mRNA and to 1 for undifferentiated CSC (Basal) and expressed as relative quantification (RQ). Data are mean ± SD of three different experiments. ANOVA with Newmann-Keuls' multicomparison test was performed: *= *p* < 0.05 and **= *p* < 0.001 vs CTL; $ = *p* < 0.05 and $$ = *p* < 0.001 vs Basal. **Panel D**. Quantitative RT-PCR analysis showing the expression of the stem cell markers (Oct4-A, Vimentin and Nanog) by CSC (Basal) and by B-CSC differentiated in the presence of anti-VR2 antibody (anti-VR2). The expression of stem cell markers were significantly decreased in B-CSC differentiated into endothelial cells and in B-CSC differentiated into endothelial cells in the presence of anti-VEGFR1 neutralizing Ab (anti-VR1) and in sFLK1 cells compared with basal condition. Data were normalized to GAPDH mRNA and to 1 for undifferentiated CSC (Basal) and expressed as relative quantification (RQ). Data are mean ± SD of three different experiments. ANOVA with Newmann-Keuls' multicomparison test was performed: *= *p* < 0.05 and **= *p* < 0.001 vs CTL; $ = *p* < 0.05 and $$ = *p* < 0.001 vs Basal. **Panel E**. The percentage of VEGFR2^+^ cells at different time points during endothelial differentiation was assessed in control B-CSC cells (CTL), or in the sFLK1 cells or in cells treated with 1 μM Sunitinib (S1) or with an anti-VEGFR2 neutralizing Ab (anti-VR2). Data are mean ± SD of three different experiments. Student's *t* test was performed: **p < 0.001 vs CTL. **Panel F**. VEGFR2 Tyr951 phosphorylation was detected in B-CSC cells expressing an empty vector (CTL), or the soluble VEGF trap sFlk1 (sFLK1 cells) by Western blot analysis of cell lysates immunoprecipitated with an anti-VEGFR2 Ab. VEGFR2 phosphorylation levels are expressed as the ratio of phosphorylated VEGFR2 to total VEGFR2. Data are representative of three different experiments.

To further investigate the role of VEGF on the differentiation process of CSC, we generated B-CSC transfected to stably express the soluble VEGF-trap sFlk1 (sFLK1 cells, Figure 4B) to obtain a constant inhibition of endogenous/exogenous VEGF binding. As found for Bevacizumab, sFLK1 cells were able to acquire endothelial markers during differentiation both at protein and mRNA level (Figure 4A and 4C) while they lost stem cell markers in respect to undifferentiated CSC (Figure 4D).

To test the role of VEGF intracellular pathway on endothelial differentiation, we inhibited the activation of VEGFRs using the anti-VEGFR2 or the anti-VEGFR1 neutralizing Abs. The anti-VEGFR2 neutralizing Ab (0.2 μg/ml) but not the anti-VEGFR1 neutralizing Ab (0.2 μg/ml) or an irrelevant Ab (not shown), administered to CSC during hypoxic endothelial differentiation, limited the acquisition of endothelial markers on CSC (Figure 4A and 4C). The lack of endothelial differentiation by treatment with the anti-VEGFR2 Ab, but not with the anti-VEGFR1 Ab, was confirmed by a concomitant maintenance of the expression of the stem markers Oct4-A, Vimentin and Nanog (Figure 4D). VEGFR2 expression remained very low in cells treated with the anti-VEGFR2 antibody, but not with the VEGF trap sFlk1, during the whole process of differentiation (Figure 4E). These data suggest that CSC endothelial differentiation may occur independently by VEGF. On the contrary, VEGFR2 activation appears relevant for this process. This was confirmed by the phosphorylation of VEGFR2 in endothelial differentiated CSC both in control and in the presence of VEGF blockade (sFLK1 cells, Figure 4F). These results suggest that interference with VEGFR2, but not with VEGFR1 or with VEGF itself, inhibits the endothelial differentiation of CSC, which is possibly due to an intracellular VEGFR2 trans-activation.

To test the relevance of intracellular pathways possibly inhibited by the anti-angiogenic treatment, we evaluated the role of the Akt pathway. A significant activation of the Akt pathway was observed in CSC under endothelial differentiation, as early as at day 3, and maintained up to complete differentiation (14 days) (Supplementary Figure 1A and 1B). However, no significant modulation of Akt activation was detected in cells treated with Sunitinib or anti-VEGFR2 or anti-VEGFR1 Abs (Supplementary Figure 1C and 1D).

### Sunitinib but not Bevacizumab impaired the hypoxia-induced HIF-1 alpha activation

**Figure 5:**
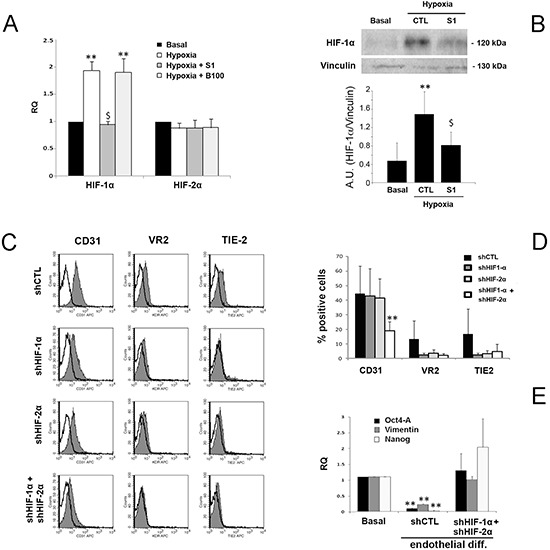
Effect of Sunitinib on HIF pathway during CSC endothelial differentiation **Panel A**. Quantitative RT-PCR analysis showing the increase of HIF-1 alpha but not of HIF-2 alpha by B-CSC incubated for 3 hours in endothelial-differentiating condition in hypoxia in the presence and absence of 1 μM Sunitinib (S1) or 100 μg/ml Bevacizumab (B100). Data were normalized to GAPDH mRNA and to 1 for time 0, expressed as relative quantification (RQ) and are mean ± SD of three different experiments. Student's *t* test was performed: **= *p* < 0.001 vs Basal; $= *p* < 0.001 vs Hypoxia. **Panel B**. Western blot micrograph and densitometric analysis of HIF-1 alpha expression. Data, shown as arbitrary units, are representative of three different experiments and were normalized to vinculin expression. Student's *t* test was performed: **= *p* < 0.001 vs Basal; $= *p* < 0.001 vs CTL. **Panel C** and **D**. Expression of endothelial differentiation markers by control B-CSC infected with a scramble shRNA (shCTL) and in B-CSC lacking HIF-1 alpha (shHIF-1α), HIF-2 alpha (shHIF-2α) or both (shHIF-1α+shHIF-2α). In the representative FACS analyses, the gray filled area shows binding of the specific antibody and the dark line the isotypic control (C). In the histogram, the percentage of expression is reported (D). Data are mean ± SD of five different experiments. Student's *t* test was performed: **= *p* < 0.001 vs shCTL. **Panel E**. Quantitative RT-PCR analysis showing the reduction of the stem-cell associated markers Oct4-A, Vimentin and Nanog in shCTL cells after endothelial differentiation in respect to basal condition, but not in endothelial differentiated shHIF-1α+shHIF-2α cells. Data were normalized to GAPDH mRNA and to 1 for time 0 and expressed as relative quantification (RQ). Data are mean ± SD of three different experiments. Student's *t* test was performed: **= *p* < 0.001 vs Basal.

As in our experimental setting endothelial differentiation required hypoxia, we also evaluated the effect of Sunitinib and Bevacizumab on the HIF pathway. When B-CSC were incubated under hypoxia in differentiating conditions, HIF-1 alpha but not HIF-2 alpha was upregulated (Figure 5A). Sunitinib impaired HIF-1 alpha upregulation both at mRNA and protein level (Figure 5A and 5B). No effect of Bevacizumab on HIF-1 alpha mRNA was observed (Figure 5A). These data indicate the ability of Sunitinib, and not of Bevacizumab, to block the hypoxia-induced intracellular pathways required for endothelial differentiation.

The requirement of HIF-1 alpha activation during the process of endothelial differentiation of B-CSC under hypoxia was confirmed using cells knocked-down for HIF-1 and/or 2 alpha. HIF-1 alpha and HIF-2 alpha double knocked-down CSC were generated since a compensatory mechanism of upregulation of HIF-1 alpha or HIF-2 alpha was observed in cells silenced for the other HIF isoform under hypoxia, (Supplementary Figure 2A and 2B). Indeed, CSC knocked down for both HIF-1 or 2 alpha significantly reduced their endothelial differentiation (Figure 5C and 5D). A partial reduction was observed in HIF-1 or 2 alpha single knock down (Figure 5C and 5D). In addition, HIF-1/2 alpha negative CSC maintained the CSC markers Oct4-A, Vimentin and Nanog, lost by control cells during endothelial differentiation (Figure 5E).

### Effect of Sunitinib and sFlk on endothelial differentiation of CSC *in vivo*

**Figure 6:**
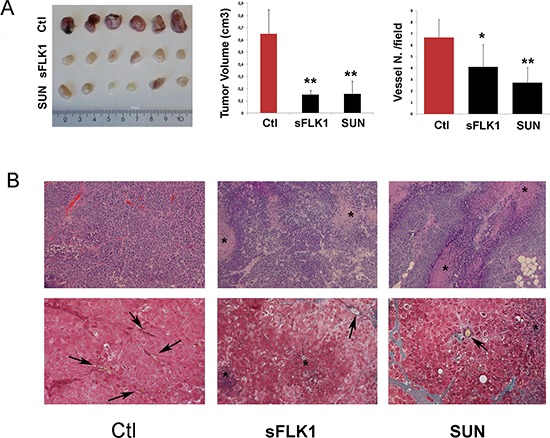
Effect of sFlk1 and Sunitinib on tumor growth and vascularization Tumors were generated by subcutaneous implant of B-CSC carrying an empty vector (Ctl) or a sFlk1 vector (sFLK1) (*n* = 8 per experimental group). Selected animals carrying Ctl tumors were treated daily with Sunitinib (SUN) from day 4. **Panel A**. Reduction of tumor volume and vascularization in sFLK1 and SUN tumors in respect to Ctl. Vessels quantification is the mean ± SD erythrocyte containing structures/field in at least 10 fields per tumor. Student's *t* test was performed: **= *p* < 0.001, *= *p* < 0.05 vs Ctl. **Panel B**. Representative micrographs of tumor sections stained with hematoxylin and eosin (upper panels) or with Masson's trichromic reaction (blu: connective, red: cells, yellow: erythrocytes; lower panels). The star indicated necrotic areas and the arrows vessels. Original magnification: H/H: 100x; Trichromic: 200x.

**Figure 7:**
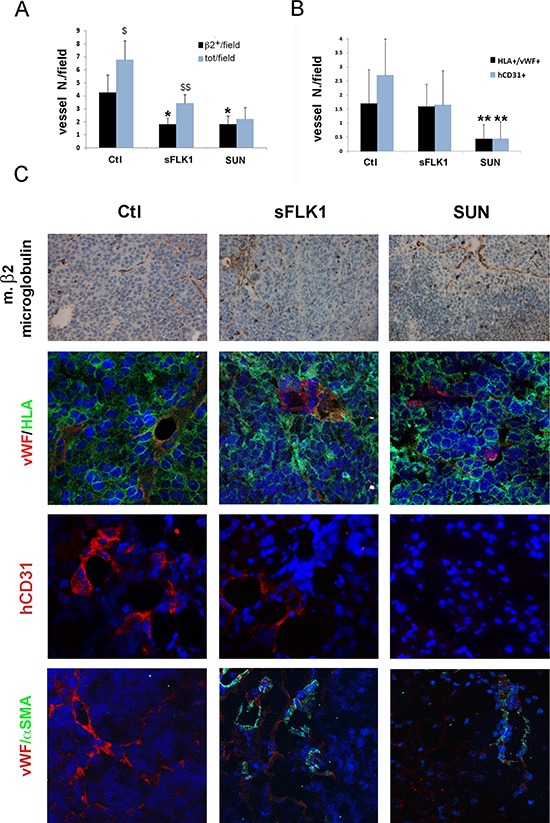
Effect of sFlk1 and Sunitinib on murine and human tumor vessels **Panel A**. Both sFlk1 releasing tumors (sFLK1) and Sunitinib treated tumors (SUN) showed reduction of murine vessels in respect to control tumors (Ctl), evaluated as murine β2-microglobuline-positive structures by immunohistochemistry (*n* = 8 per experimental group). Murine vessels only represented a part of total vessels in Ctl and sFLK but not in SUN tumors. Data are the mean ± SD of β2-microglobulin positive structures containing erythrocytes (β2^+^)/field or total erythrocytes containing structures in at least 10 fields per tumor. Student's *t* test was performed: **= *p* < 0.001, *= *p* < 0.05 vs Ctl; $$= *p* < 0.001, $= *p* < 0.05 vs β2^+^/field. **Panel B**. SUN tumors and not sFLK1 tumors showed reduction of human vessels, evaluated as structures co-expressing vWF and human HLA Class I (vWF^+^/HLA^+^) by immunofluorescence. Data are the mean ± SD of vWF^+^/HLA^+^ structures/field in at least 10 fields per tumor. Student's *t* test was performed: **= *p* < 0.001 vs Ctl. **Panel C**. Representative micrographs of Ctl, sFLK1 and SUN tumor sections showing murine β2-microglobulin positive structures (upper panels). Representative micrographs of Ctl, sFLK1 and SUN tumor sections showing positive immunofluorescence staining for vWF (red) /human HLA class-I (green) positive structures, for human CD31 (hCD31, red) or for vWF (red)/αSMA (green). Original magnification: x200 (upper panels) and x400 (lower panels).

CSC injected subcutaneously in SCID mice organized after 7 days in small clusters of few cells and, after 14 days, in highly vascularized tumors (Supplementary Figure 3A and 3B and Figure 6A and 6B). At an early tumor phase, vessels were mainly of murine origin, as endothelial cells did not express HLA (Supplementary Figure 3C and 3D). At variance, in large tumors, some intratumor vessels were of human origin, as detected by human HLA Class I and vWF co-expression (Figure 7B and 7C). This is possibly related to the detection of hypoxic areas in large tumors that showed a strong expression of the hypoxic marker carbonic anhydrase IX (CAIX). Small tumor clusters did not express CAIX (Supplementary Figure 3E and 3F). We therefore evaluated the possible differential effect of VEGF or tyrosine kinase blockade on endothelial differentiation of CSC *in vivo* after 14 days. SCID mice were treated as follows: (i) mice injected subcutaneously with B-CSC expressing the soluble VEGF trap sFlk1 (sFLK1 mice), (ii) mice injected subcutaneously with B-CSC transduced with an empty vector as control (Ctl mice), (iii) mice injected subcutaneously with control B-CSC and daily treated with oral administration of Sunitinib (50 mg/kg, SUN mice). Tumors generated by sFLK1 cells, in which the released sFlk1 is able to sequester human and murine VEGF, showed reduced growth and vascularization and extensive necrosis (Figure 6A and 6B). The analysis of the vessels showed co-existence of both β2-microglobulin^+^ murine vessels and vWF^+^/HLA^+^ human vessels (Figure 7A–7C). In SUN mice, tumors generated by B-CSC showed reduced growth and vascularization and extensive necrosis comparable to that in sFLK1 mice (Figure 6A and 6B). The murine vessels in SUN tumors were reduced in respect to Ctl to an extent similar to sFLK1 tumors. However, the number of human vessels detected was almost negligible, and the great percentage of vessels observed was of murine origin (Figure 7A–7C). These data indicate that *in vivo* endothelial differentiation of B-CSC, i.e. vasculogenesis, is independent of VEGF inhibition by sFlk1, but dependent on endothelial receptor tyrosine blockade by Sunitinib. Finally, we evaluated the presence of pericytes around the vessels of treated tumors, as a sign of vascular stability. In SUN and sFLK1 tumors, the few vessels detectable were covered by α-SMA^+^ (Figure 7C) cells. At variance, vessels detected in Ctl mice within the tumor were mainly negative (Figure 7C). These results altogether may suggest that the anti-angiogenic treatment using both Sunitinib and VEGF blockade reduces tumor vascularization while it stabilizes the surviving vessels, as reported [19]. Moreover, Sunitinib could specifically block tumor CSC-dependent vasculogenesis.

## DISCUSSION

In the present study, we investigated the effect of drugs targeting the VEGF pathway on tumor vasculogenesis. The results show a differential effect of anti-VEGF drugs and Sunitinib on CSC-induced vasculogenesis. VEGF blockade only affected differentiated endothelial cells *in vitro* and murine angiogenesis *in vivo*, respectively. At variance, Sunitinib also impaired the process of differentiation of CSC into endothelial cells *in vitro* and the CSC-induced vasculogenesis *in vivo*.

Tumor vascularization is granted by combined mechanisms of angiogenesis and vasculogenesis, and anti-tumor therapies should ideally affect both. We here found that Sunitinib and Bevacizumab displayed a cytotoxic and anti-proliferative response on endothelial cells derived from CSC that was comparable to the response of the total endothelial tumor population. A similar inhibitory effect was obtained by VEGF-blockade (sFlk1) and Sunitinib that affected the murine vessels originated in CSC-induced tumors *in vivo*. These data indicate that both VEGF blockade and Sunitinib can target differentiated endothelial cells generated either by vasculogenesis or angiogenic mechanisms. As previously reported for tumor-derived endothelial cells [20], the sensitivity to anti-angiogenic drugs of CSC-derived endothelial cells was lower than that of normal endothelial cells, underlying possible mechanisms of drug resistance.

In this study, we also evaluated the effect of anti-angiogenic drugs on CSC endothelial differentiation in hypoxic condition, as CSC were mainly located in an hypoxic perivascular niche, in close association to tumor vessels [21], where hypoxia may promote CSC differentiation [10, 22]. Interestingly, we found a differential effect of drugs acting through VEGF receptor blockade (Sunitinib and anti-VEGFR2 blocking antibody) in respect to those acting through VEGF inhibition (Bevacizumab and the VEGF soluble trap sFlk1) on CSC differentiation. Only Sunitinib, and not Bevacizumab or sFlk1, inhibited the ability of CSC to differentiate into endothelial cells *in vitro* or to organize into vessels *in vivo*. A similar effect was observed with VEGFR2 and not VEGFR1 inhibition underlying the importance of the VEGFR2 receptor activation. Several possibilities may explain this difference between VEGFR2 and VEGF blockade on CSC differentiation. For instance, VEGFR2 might be trans-activated at an intracellular level, possibly by lipid metabolites, cytokine receptors and Plexin-A4 [23–27]. Similarly, we found that VEGFR2 was phosphorylated in endothelial differentiated CSC even when VEGF was inhibited by the constant release of the sFlk1 VEGF trap, suggesting a possible role for VEGFR2 activation in the absence of VEGF binding.

The effect of Sunitinib could also be explained by targeting of different VEGF-unrelated receptor tyrosine kinases such as CD140, CD117, and RET [28]. However, its activity in renal and breast CSCs appears dependent on VEGFRs, as the others receptors are absent in both undifferentiated and endothelial differentiated CSC. Finally, Sunitinib may directly act on intracellular targets, such as Akt or HIF. In colon cancer cells, Sunitinib was shown to inhibit the HIF-1 alpha translation accompanied with inactivation of Akt, possibly suggesting that Sunitinib may directly act on Akt [30, 31]. However, no effect on Akt activation was observed in our setting. In addition, in neuroblastoma cells, Sunitinib was reported to block HIF activation independently of receptor tyrosine kinase inhibition [32]. We similarly found that HIF-1 alpha synthesis, required for CSC endothelial differentiation under hypoxia, was inhibited by Sunitinib, although we cannot distinguish between a direct or a cytokine-dependent effect. In fact, HIF may be activated both directly and indirectly by tyrosine kinase-dependent intracellular pathways [29].

Regardless the mechanism involved, the specific role of Sunitinib on CSC endothelial differentiation supports its role in tumor therapy. Indeed, although Sunitinib did not directly affect CSC, it blocked both the angiogenesis and the hypoxia-driven CSC vasculogenesis, leading to tumor necrosis and inhibition of its development. On the other side, some possible negative consequences of the observed effects of Sunitinib can be envisaged. The first is a role in the maintenance of an undifferentiated CSC population, as shown by the maintenance of stem cell markers by Sunitinib treated CSC. Indeed, it was recently described that Sunitinib may promote embryonic stem cell self-renewal and limit their differentiation, even in the presence of established differentiating factors [33]. In addition, tumor hypoxia possibly induced by anti-angiogenic therapy itself may sustain mechanisms of adaptation that promote tumor progression to greater malignancy or increased tumor cell invasion [34–37].

In conclusion, our data indicate that VEGF inhibition may only affect fully differentiated endothelial cells, while Sunitinib, possibly through VEGFR blockade, is also able to impair CSC-dependent tumor vasculogenesis under hypoxia. As hypoxia induced by the anti-angiogenic therapy itself may promote tumor angiogenesis and vasculogenesis, the limitation of the mechanisms of CSC endothelial differentiation are required for its efficacy.

## METHODS

### Cancer stem cell isolation and characterization

Breast cancer stem cells (B-CSC) and renal cancer stem cells (R-CSC) were isolated, cloned, and characterized as previously described [15, 16; 38–41; Supplementary Table 1]. Briefly, B-CSC were obtained from a specimen of a lobular-infiltrating carcinoma obtained from a consenting patient according to the Ethics Committee of the S. Giovanni Battista Hospital of Torino, Italy, as previously described [16]. B-CSC were isolated and expanded as mammospheres in 10 ng/ml basic fibroblast growth factor (bFGF), 20 ng/ml epidermal growth factor (EGF), 5 μg/ml insulin and 0.4% bovine serum albumin (all from Sigma-Aldrich), as previously described [40]. R-CSC were obtained from specimens of renal carcinomas from patients undergoing radical nephrectomy. CD105^+^ R-CSC were cultured in the presence of the expansion medium, a modification of that described for multipotent adult progenitor cells [42], consisting of DMEM LG (Invitrogen), with insulin-transferrin-selenium, 10^−9^ M dexametasone, 100 U penicillin, 1000 U streptomycin, 10 ng/ml EGF (all from Sigma-Aldrich) and 5% fetal calf serum (FCS) (Sigma-Aldrich). A CD105^+^ clonal renal cancer stem cell line was selected and used for all the experiments. B-CSC and R-CSC were characterized as tumor stem cells due to the following criteria, previously described for cancer stem cells present in other tumor types [43]: *1*) were clonogenic, *2*) expressed stem cell markers and lacked differentiative markers, *3*) could differentiate *in vitro* into epithelial and endothelial cell types, and *4*) could generate *in vivo* serially transplantable tumors. These tumors, despite being derived from clones expressing mesenchymal markers, were epithelial carcinomas as the tumor of origin [15, 16, 39; Supplementary Table 1].

Human umbilical vein endothelial cells (HUVEC) were obtained and characterized as previously described [44]. A tumor endothelial cell line (BTEC) obtained from a breast tumor was previously isolated and characterized [45].

### Anti-angiogenic drugs

Sunitinib malate (Sigma-Aldrich) and Bevacizumab (Genentech) were stocked according to the manufacturer's instructions.

### Epithelial and endothelial differentiation of CSC *in vitro*

Epithelial differentiation was obtained in the presence of RPMI plus 10% FCS, without the addition of growth factors, as previously described [15, 16]. For endothelial differentiation, B-CSC and R-CSC were plated into 6-well culture plated coated with Endothelial Cell Attachment Factor (Sigma-Aldrich), in Endogro (Merck Millipore) without growth factor supplement and maintained in hypoxia (1% O_2_ and 5% CO_2_) in hypoxia chambers (Stem Cells Technologies) for 14 days. The anti-angiogenic drugs (Sunitinib 1 μM, Bevacizumab 100 μg/ml) and the anti-VEGFR1 or anti-VEGFR-2 blocking polyclonal Ab, or an irrelevant rabbit serum (all from R&D Systems), all at 0.2 μg/ml, were added to cell cultures under hypoxic differentiation at day 0 and every three days thereafter.

### Proliferation and survival

DNA synthesis was detected as incorporation of 5-bromo-2-deoxyuridine (BrdU) using an enzyme-linked immunosorbent assay kit (Chemicon) after 48 hours of treatment. To evaluate cell death, supernatants containing detached and death cells were and cells were trypsinized. Cell suspensions were stained with 100 μg/ml propidium iodide in PBS containing 0.1% Triton. Cell cycle distribution was determined using a Beckton Dickinson FACScan flow cytometer, analysing 10000 cells per sample, and the percentage of cells in sub-G1 phase (apoptotic cells) was estimated.

### Tubulogenesis

*In vitro* formation of capillary-like structures was done on growth factor–reduced Matrigel (BD Biosciences). After hypoxia-induced endothelial differentiation, cells (3 × 10^4^ cells per well) were seeded onto Matrigel-coated wells in RPMI plus 5% FCS with or without Sunitinib or Bevacizumab. Cells were periodically observed with a Nikon inverted microscope and experimental results recorded after 18 hours. Image analysis was performed with the MicroImage analysis system (Cast Imaging srl).

### Western blot analysis

Cells were lysed in RIPA buffer supplemented with protease and phosphatase inhibitor cocktail and PMSF (Sigma-Aldrich). Aliquots of the cell lysates containing 50 μg protein, as determined by the Bradford method, were run on 8% SDS-PAGE under reducing conditions and blotted onto PVDF membrane filters using the iBLOT system (Life Technologies). For Western blot analysis, anti-HIF-1 alpha, anti-vinculin, anti-actin (all from Santa Cruz Biotechnology), anti-AKT or anti p-AKT(Ser473) (both from Cell Signalling) Abs were used. sFlk1 expression was tested in the supernatant of B-CSC maintained in culture in absence of serum for 24 h. After centrifugation to remove the cell debris, cell-free supernatants were concentrated 25-fold by centrifugation using Ultra-PL 3 ultrafiltration units (Amicon-Ultra, Millipore) with a 3-kDa molecular weight cut off and Western blot performed using the anti-Flk1 Ab (R&D Systems). For immuonoprecipitation studies, 10 μg of anti-Flk1 antibody (R&D Systems) was coupled with 1.5 mg Dynabeads-Protein G (Life Technologies) in order to precipitate 1 mg of cell lysates, according to manufacturer's instructions. The immunoprecipitated samples were immediately processed for electrophoresis and Western blot analysis, using anti-VR2 (R&D Systems) and anti p-VR2 (Tyr951) (Santa Cruz Biotechnology). Differences in protein phosphorylation were evaluated as VR2/p-VR2 (Tyr951) ratio.

### RNA isolation and real time PCR

Total RNA was isolated using Trizol Reagent (Ambion) according to the manufacturer's protocol, and quantified spectrophotometrically (Nanodrop ND-1000). For gene expression analysis, quantitative real-time PCR was performed. Briefly, first-strand cDNA was produced from 200 ng of total RNA using the High Capacity cDNA Reverse Transcription Kit (Applied Biosystems).

Quantitative Real-time PCR experiments were performed in 20-μl reaction mixture containing 5 ng of cDNA template, the sequence-specific oligonucleotide primers (purchased from MWG-Biotech) and the Power SYBR Green PCR Master Mix (Applied Biosystems). GAPDH was used to normalize RNA inputs. Fold change expression respect to control was calculated for all samples. The sequence-specific oligonucleotide primers are available in the Supplementary Materials.

### Generation of HIF^−/−^ CSC and of sFLK1 CSC

For knock down of HIF-1 alpha and HIF-2 alpha, a pGIPZ lentiviral vector (Open Biosystems) carrying shRNA against HIF-1 alpha, HIF-2 alpha or scramble was used (see Supplementary Materials). The constructs were then transfected with the 293T cell line using the ViraPower Packaging Mix (Life Technologies) for lentiviruses production. After titering the lentiviral stock, CSC were transduced with lentiviral particles following the manufacturer's instructions. Cells were selected by puromycin (Gibco) (250 ng/ml) and antibiotic-resistant cells were expanded. Cell infection was evaluated by GFP^+^ > 90%, as assessed by FACS analysis, and by down regulation of the target gene > 60% by quantitative RT-PCR. CSC silenced for HIF-1 or 2 alpha significantly reduced both HIF isoforms as compared to control cells (Supplementary Figure 2).

For the generation of sFLK1 B-CSC, we used a lentiviral vector carrying the sequence of the soluble form of the VEGFR2 receptor (sFlk1) under the control of the CMV promoter, and an empty vector as a control as described [29]. Cell infection was evaluated by the presence of the soluble form of VEGFR2 on the supernatant of B-CSC by western blot (Figure 4B).

### *In vivo* experiments

To evaluate the vasculogenic potential of B-CSC, 4 × 10^5^ cells were implanted subcutaneously into SCID mice (Charles River) within Growth Factor–Reduced Matrigel (BD Biosciences). The effect of VEGF blockade on vasculogenesis was studied by using B-CSC infected with a soluble Flk1 lentivirus (sFLK1 cells), able to bind both human and murine VEGF (Figure 4B). Briefly, 4 × 10^5^ cells infected with lentiviruses carrying the empty vector (Ctl) or the sFlk1 vector (sFLK1) were resuspended in 50 μl DMEM plus 150 μl of Matrigel at 4°C. Cells were injected subcutaneously into the left back of SCID mice (*n* = 12 for the Ctl group, *n* = 8 for the sFLK1). In addition, a group of animals injected with 4 × 10^5^ cells infected with lentiviruses carrying the empty vector (Ctl) were orally treated with Sunitinib (50 mg/kg) starting from day four after cell injection (*n* = 8). After 7 or 14 days, mice were sacrificed, and tumors recovered and processed for histology.

### Immunohistochemistry and immunofluorescence

Sections from paraffin-embedded blocks of tumors obtained from SCID mice were stained with hematoxilyn and eosin or with Trichromic Masson reaction (BioOptica) to detect vessels, or with an anti-mouse β2-microglobulin Ab, (Santa Cruz), or with anti-Carbonic Anhydrase IX (CAIX) Ab (Novus Biologicals). At least 10 pictures/tumor were taken at a 200X magnification. Vessel count was performed in blind on Masson's trichromic or murine β2-microglobulin stained sections and expressed as number of structures with red blood cells/field. Immunofluorescence was performed on cells cultured on chamber slides (Nunc) using the following Abs: anti-von Willebrand Factor (vWF) Ab (Dako), anti-pan-cytokeratin (CK) Ab (Biomeda), VE-Cadherin (VE-Cad) Ab (Merck Millipore). Immunofluorescence was also performed on cryostatic sections for HLA-class I Ab, for vWF (BioLegend), human CD31 (Becton Dickinson) and α-SMA (Sigma-Aldrich). Alexa Fluor488 or Texas Red-conjugated anti-rabbit and anti-mouse IgG (Molecular Probes) were used as secondary Abs. Hoechst 33258 dye (Sigma-Aldrich) was added for nuclear staining. Confocal microscopy analysis was performed using a Zeiss LSM 5 Pascal model confocal microscope (Carl Zeiss). For cytofluorimetric analysis cells were stained with the following fluorescein isothiocyanate, phycoerythrin or allophycocyanin-conjugated antibodies: CD24, CD44, CD140, CD31 (all from Becton Dickinson) CD105, VEGFR2 (both from Miltenyi Biotec), CD117 (Dako), TIE2, VEGFR1, VEGFR3 (all from R&D Systems). Isotypes (Miltenyi Biotec) were used as negative controls. Cells were subjected to cytofluorimetric analysis (FACScan Becton Dickinson).

### Statistical analysis

Statistical analysis was performed by using the Student *t* test, or ANOVA with Dunnet's or Newmann Keuls' multicomparison tests, as appropriate. A *p* value of < 0.05 was considered significant.

## SUPPLEMENTARY MATERIALS, FIGURES AND TABLE


