# Supplementary material for: Sunitinib but not VEGF blockade inhibits cancer stem cell endothelial differentiation
Source: Oncotarget. 2015 Feb 28;6(13):11295–309. doi: 10.18632/oncotarget.3123 (PMC4484457; doi:10.18632/oncotarget.3123)
Supplement: Supplementary file 1 [file oncotarget-06-11295-s001.pdf]

SUPPLEMENTARY MATERIALS

RNA isolation and Real time PCR

Total RNA was isolated from different cell preparations using Trizol Reagent (Ambion) according to the manufacturer's protocol. RNA was then quantified spectrophotometrically (Nanodrop ND- 1000). For gene expression analysis, quantitative real-time PCR was performed. Briefly, first-strand cDNA was produced from 200 ng of total RNA using the High Capacity cDNA Reverse Transcription Kit (Applied Biosystems).

Quantitative Real-time PCR experiments were performed in 20-µl reaction mixture containing 5 ng of cDNA template, the sequence-specific oligonucleotide primers (purchased from MWG-Biotech) and the Power SYBR Green PCR Master Mix (Applied Biosystems). GAPDH was used to normalize RNA inputs. Fold change expression respect to control was calculated for all samples. The sequence-specific oligonucleotide primers used are GAPDH: forward, 5′-TGGAAGGACTCATGACCACAG T-3′ and reverse, 5′-CATCACGCCACAGTTTCCC-3′; KDR: forward 5′-GAACATTTGGGAAATCTCTTGCA-3′, and reverse 5′-AGTCCAGAATCCTCTTCCATGCT-3′; TIE-2 forward 5′-CCCCTATGGGTGTTC-3′, and reverse GCTTACAATCTGGCC-3′; HIF-1 alpha: forward, 5′-TGCACTCAATCAAGAAGTTGCA-3′, and reverse, 5′-GGACTATTAGGCTCAGGTGAACTTTG-3′; HIF-2 alpha: forward, 5′-TGACCCAAGATGGCGACA T-3′, and reverse 5′-CTCCACCTGTGTAAGTCCCATGA-3′; OCT4-A: forward, 5′-AGCAGGAGTCGGGGTGG-3′ and reverse, 5′-CTGGGACTCCTCCGGGTT-3′; NANOG: forward, 5′-ACAACTGGCCGAAGAATAGCA-3′ and reverse, 5′-GGTTCCCAGTCGGGTTCAC-3′; VIMENTIN: forward, 5′-GGAACAGCATGTCCAAAT CGA T-3′, and reverse, 5′-CAGCAAACTTGGATTTGTACCATT-3′.

Generation of HIF$^{-/-}$ CSC

For knock down of HIF-1 alpha and HIF-2 alpha, a pGIPZ lentiviral vector (Open Biosystems) carrying shRNA against HIF-1 alpha (CCGACACAGCCTGGATATGAAATAGTGAAGCC AC AGATGTATTTCATATCCAGGCTGTGT), HIF-2 alpha (ACCACCTTCAATGACTTCTAATTAGTGAAGCCAC AGATGTAATTAGAAGTCATTGAAGGTGGG) or scramble (GATCCCCAGATCTCAAGTTCCTCACATT CAAGAGATGTGAGGAACTTGAGATCTTTTTAAG CT) was used. The constructs were then transfected with the 293T cell line using the ViraPower Packaging Mix (Life Technologies) for lentivirus production. After titering the lentiviral stock, CSC were transduced with lentiviral particles following the manufacturer's instructions. Cells were selected by puromycin (Gibco) (250 ng/ml) and, after 6 days, antibiotic-resistant cells were expanded. Cell infection was evaluated by GFP+ > 90%, as assessed by FACS analysis, and by down regulation of the target gene > 60% by quantitative RTPCR. CSC silenced for HIF-1 or 2 alpha significantly reduced both HIF isoforms as compared to control cells (Supplementary Figure 1).

SUPPLEMENTARY FIGURES AND TABLE

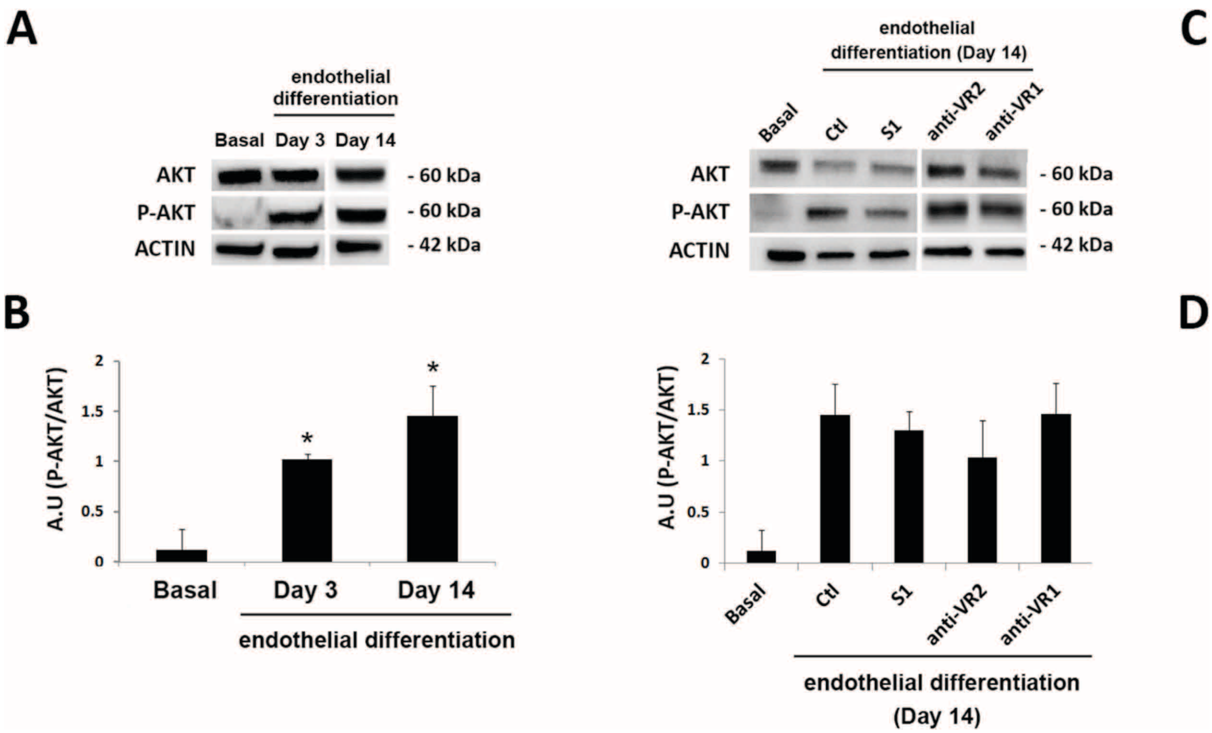

Supplementary Figure 1: Effect of Sunitinib and VEGFR inhibition on AKT phosphorylation in endothelial differentiated CSC. **Panel A** and **B**. Western blot analysis and relative quantification (A. U.) of basal Akt and phosphorylated Akt (Ser473) (P-AKT) in undifferentiated B-CSC (Basal) and in B-CSC differentiated in endothelial cells at day 3 and 14. Expression of phospho-Akt/Akt ratio normalized to actin. Values are expressed as mean ± SD of two independent experiments. Student's *t* test was performed: *= $p < 0.05$. **Panel C** and **D**. Western blot analysis and relative quantification (A. U.) of basal Akt and P-Akt in undifferentiated B-CSC (Basal) and in endothelial differentiated B-CSC untreated (Ctl), or treated with 1 µM Sunitinib (S1) or anti-VEGFR2 (anti- VR2) or anti- VEGFR1 (anti-VR1) Abs. Expression of phospho-Akt/Akt ratio normalized to actin. Values are expressed as mean ± SD of two independent experiments.

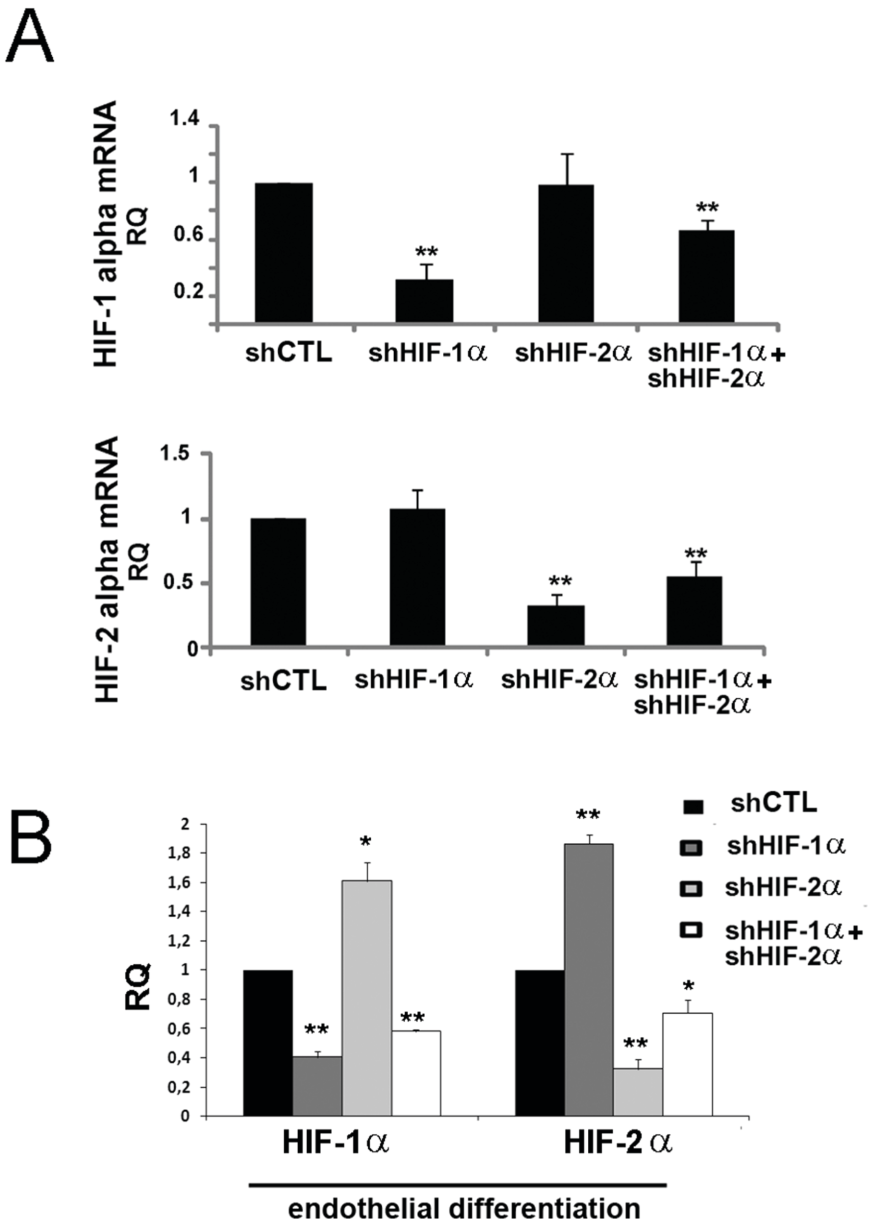

Supplementary Figure 2: Effect of HIF pathway inhibition on the endothelial differentiation of B-CSC. Panel A and **B**. Quantitative RT-PCR analysis showing the expression of HIF-1 alpha and HIF-2 alpha mRNA in control B-CSC cells infected with a scramble shRNA (shCTL) and in B-CSC lacking HIF-1 alpha (shHIF-1α), HIF-2 alpha (shHIF-2α) or both (shHIF-1α+shHIF-2α) in basal conditions (A) and after endothelial differentiation (B). Data were normalized to GAPDH mRNA and to 1 for time 0. Data are mean ± SD of three different experiments. Student's *t* test was performed: **= $p < 0.001$, *= $p < 0.05$ vs shCTL.

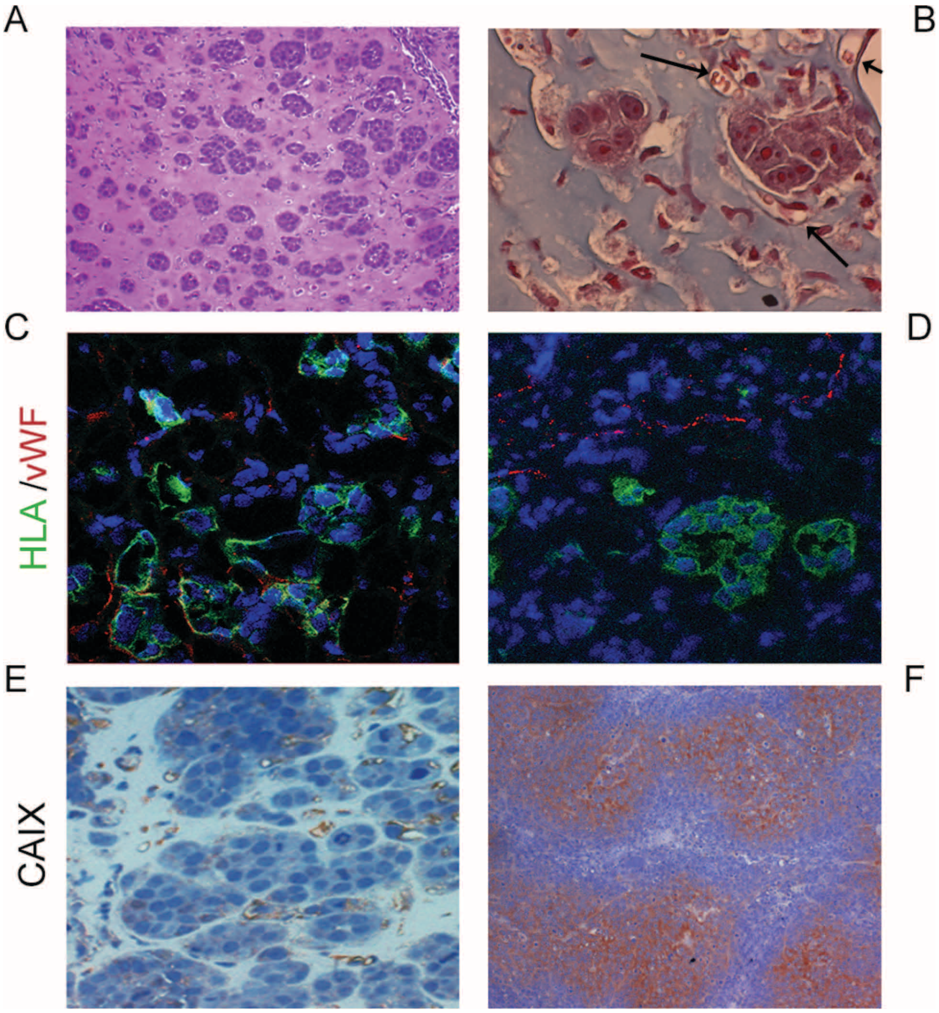

Supplementary Figure 3: Early phase of *in vivo* tumorigenesis by B-CSC. **Panel A** and **B**. Representative micrographs of CSC-generated tumors (*n* = 4) 7 days after cell injection (4X105 cells). Small clusters of tumor cells (A) and small vessels within the matrix (arrows, B) were detectable (A: Hematoxylin and Eosin, B: Masson's trichromic reaction). **Panel C** and **D**. Immunofluorescence images showing human HLA class I+ (green) tumor cells and vWF+ (red) endothelial cells. HLA negative vessels were detected around tumor clusters (C) and within matrix (D). **Panel E** and **F**. Representative micrographs showing immunohistochemical detection of the hypoxic marker CAIX in tumors generated after 7 days (E) and 14 days (F). Areas of hypoxia were present only in late tumors. Original magnification: A, B, E and F: 200x, C and D: 400x

Supplementary Table 1: Characteristics of CSC derived from renal and breast carcinomas

	R-CSC	B-CSC
**Clonogenic ability**	+	+
**Stem cell marker expression**	Nanog, Musashi, Nestin, Oct-4	Nanog, Nestin, Oct-4
**Mesenchymal marker expression**	CD105, CD44, CD90, CD146, CD73, CD29, Vimentin	CD105, CD44, CD90, CD146, CD73, CD29, Vimentin
**Epithelial marker expression**	Absent	Absent
**Epithelial differentiation**	*In Vitro:* CK7, Pan-CK, E-Cadherin	*In Vitro:* CK18, CK14, Pan-CK
	In Vivo: Epithelial tumors resembling the tumor of origin	*In Vivo:* Epithelial tumors resembling the tumor of origin
**Endothelial differentiation**	*In Vitro:* vWF, VEGFR1, VEGFR2, VEGFR3, CD31, Tie-2, VE-Caderin	*In Vitro:* vWF, VEGFR1, VEGFR2, VEGFR3, CD31, Tie-2, VE-Caderin
	In Vivo: HLA^+ vessels	*In Vivo:* $HLA^+/hCr17^+$ vessels,
**Tumor initiating ability**	+	+
**Serially transpantable tumors**	+	Not done
**References**	[15, 39]	[16, 43]

Abbreviations: R-CSC: renal cancer stem cells; B-CSC: breast cancer stem cells; HLA: Human leukocyte antigen, hCr17: human chromosome 17.
